# Increased Intra-Individual Variability of Cognitive Processing in Subjects at Risk Mental State and Schizophrenia Patients

**DOI:** 10.1371/journal.pone.0078354

**Published:** 2013-11-08

**Authors:** Ye Seul Shin, Sung Nyun Kim, Na Young Shin, Wi Hoon Jung, Ji-Won Hur, Min Soo Byun, Joon Hwan Jang, Suk Kyoon An, Jun Soo Kwon

**Affiliations:** 1 Department of Brain and Cognitive Sciences – World Class University Program, College of Natural Sciences, Seoul National University, Seoul, Republic of Korea; 2 Department of Psychiatry, Seoul National University College of Medicine, Seoul, Republic of Korea; 3 Department of Neuropsychiatry, Seoul National University Hospital, Seoul, Republic of Korea; 4 Institute of Human Behavioral Science, SNU-MRC, Seoul, Republic of Korea; 5 Department of Psychiatry, Yonsei University College of Medicine, Seoul, Republic of Korea; Baylor College of Medicine, United States of America

## Abstract

Intra-individual variability (IIV) has received recent attention as an indicator of the stability of cognitive functioning that may outperform mean performance in reflecting putative neurobiological abnormalities. Increased IIV is regarded as a core deficit in schizophrenia patients; however, whether this deficit is present in the prodromal phase before the onset of schizophrenia has not been well established. In the present study, we investigated IIV using the stop-signal paradigm in at-risk mental state (ARMS) individuals and in schizophrenia patients. The study included 27 ARMS subjects, 37 schizophrenia patients, and 38 normal controls. The stop-signal task was administered to assess IIV and response inhibition. IIV was estimated by calculating the standard deviation across sub-blocks for the three groups. We observed increased IIV in ARMS subjects and schizophrenia patients compared with normal controls in both the “stop” and the “go” processes even though the mean response inhibition performances were not impaired in the ARMS group. Schizophrenia patients showed impaired response inhibition that was associated with the severity of negative symptoms. Our findings suggest that the analysis of IIV may identify cognitive and clinical features of ARMS that are not detectable by conventional mean performance analysis. The unstable response patterns associated with ARMS may originate from abnormal processing in neural systems caused by alterations in the integrity of functional brain networks and dopamine neuromodulation.

## Introduction

The last decade has witnessed increasing interest in the prodromal states of schizophrenia and at-risk mental states (ARMS) and focused on early intervention to delay or prevent the onset of psychosis [1]. Neuropsychological findings have demonstrated deficits in several cognitive domains, including working memory [2], attention [3], social functioning [4], and executive function [2], [3], that are apparent prior to the onset of the illness [5]. However, neurocognitive studies of ARMS subjects have commonly relied on examination of their average performance. Although mean measures are useful as indices for capturing cognitive performance, emphasizing the mean may overlook other important facets of cognitive functioning [6] and lead to erroneous inferences [7]. Specifically, when within-person variability increases, the calculation of mean performance from single measurements may lead to a poor estimation of group differences [8].

Intra-individual variability (IIV) is a measure of short-term fluctuations in an individual's performance and is regarded as an indication of the stability of cognitive processing and not simply as uninformative noise [9]. IIV provides information regarding cognitive functioning that is not detectable by average measures of performance [10] and can better discriminate cognitively impaired and clinical groups from normal controls [11]. Accumulating evidence indicates that IIV reflects alterations that occur at the neural level of the brain [12], [13] and thus may be a useful early index of underlying brain pathology [14]. In particular, frontal lobe circuitry is associated with IIV, which reflects a greater demand for executive control processes to maintain task performance [15], [16], [17], [18]. Increased IIV has been reported in patients with frontal lobe dysfunctions, such as schizophrenia [16], [19], [20], [21], ADHD [22], [23], and traumatic brain injury [17]. In addition, alterations in dopamine (DA) neuromodulation have been linked to increased IIV in several conditions including schizophrenia [24], [25], ADHD [26], and Parkinson's disease [27]. Given that ARMS subjects show abnormal frontal lobe processing [28] and alterations in DA function [29], [30], increased IIV may be present in ARMS subjects.

The aim of the present study was to examine IIV in ARMS subjects and schizophrenia patients using a response inhibition task that is related to frontal lobe functioning (i.e., a stop-signal paradigm) to determine whether increased IIV is present in the prodromal phase of schizophrenia. Furthermore, we aimed to investigate the difference between these two groups with regard to mean performance and IIV. We predicted significantly higher IIV in both ARMS subjects and schizophrenia patients compared to controls.

## Methods

### Ethics statement

This study was approved by the Institutional Review Board at Seoul National University Hospital (IRB No. H-1110–009–380), and written informed consent was obtained from all participants prior to beginning the study, including parental consent for those younger than 18 years of age.

### Participants

The sample consisted of 27 subjects with ARMS for psychosis, 37 patients with schizophrenia, and 38 normal controls. The demographic and clinical characteristics of the three groups are summarized in Table 1. The ARMS subjects were recruited from the Seoul Youth Clinic (SYC), which is currently conducting a longitudinal study of individuals who are at high risk for psychosis using criteria from the Comprehensive Assessment of At-Risk Mental States (CAARMS) [31] and the Korean version of the Structured Interview for Prodromal Syndromes (SIPS) [32], [33]. The ARMS subjects met the criteria in at least one of the following three categories: 1) attenuated psychotic symptoms (n = 27), 2) brief limited intermittent psychotic symptoms (n = 0), and 3) trait-and-state risk factors (n = 6). Six subjects were categorized with both attenuated psychotic symptoms and trait-and-state risk factors. Six of the 27 participants in the ARMS group were receiving treatment with antidepressants, benzodiazepines, or beta-blockers at the time of assessment. No ARMS subjects were receiving treatment with antipsychotics. The schizophrenia patients were recruited from the outpatient clinic at the Department of Psychiatry of Seoul National University Hospital and fulfilled the DSM-IV criteria for schizophrenia, as diagnosed using the Structured Clinical Interview for DSM-IV (SCID) [34]. Exclusion criteria were a history of traumatic brain injury, epilepsy, substance abuse, or other neurologic illness. At the initial assessment, 31 of the 37 patients in the schizophrenia group were taking antipsychotic medication. They were all receiving treatment with atypical antipsychotics, and the mean daily dose (chlorpromazine equivalents) was 387.9 mg. Two of them were also taking low-dose treatment of typical antipsychotics, for which the mean daily dose was 195 mg. The severity of the psychotic features, anxiety symptoms, and depressive symptoms of the ARMS subjects and schizophrenia patients were assessed using the Positive and Negative Syndrome Scale (PANSS) [35], a modified version of the Brief Psychiatric Rating Scale (BPRS) [36], the Hamilton Anxiety Rating Scale (HAM-A) [37], and the Hamilton Depression Rating Scale (HAM-D) [38], respectively. Participants in the control group were recruited from the community through internet advertisements and by using the Structured Clinical Interview for DSM-IV, Non-patient version (SCID-NP). Participants were screened with an additional exclusion criterion of any first- or second-degree relatives with a history of a psychotic disorder. Exclusion criteria for the ARMS and control groups were mental retardation, any lifetime diagnosis of psychiatric illness, substance abuse, a history of head injury, and neurological disorders. All groups were further assessed using the Global Assessment of Functioning (GAF) to rate overall social, occupational, and psychological functioning.

**Table 1:** Demographic and clinical characteristics.

	Control	ARMS	Schizophrenia	Statistics	
	(n = 38)	(n = 27)	(n = 37)	*χ ^2^* or *F, t*	*p-value*
Male/Female	23/15	16/11	15/22	4.28	0.118
Age (year)	22.4(2.7)	20.9(2.8)	22.6(3.8)	2.65	0.075
Education year	14.3(1.7)	13.2(2.0)	13.6(2.3)	2.53	0.084
IQ	108.4(11.4)	108.1(9.6)	98.5(12.2)	8.9	<0.001
GAF	88.6(1.9)	49.5(6.7)	49.9(10.3)	349.77	<0.001
PANSS total		66.7(14.1)	62.3(14.2)	−1.2	0.234
Positive score		14.2(2.6)	14.1(4.8)	−1.14	0.892
Negative score		18.0(6.1)	16.6(5.2)	−0.99	0.327
General score		34.5(8.6)	31.7(7.3)	−1.35	0.183
CAARMS^a^		48.3(14.2)			
HAM-D		12.0(6.1)	8.92(5.4)	−2.09	0.041
HAM-A		9.9(6.8)	6.6(4.9)	−2.09	0.042
BPRS		47.1(8.9)	43.03(8.8)	−1.81	0.076

*Note.* Data are presented as the mean (SD). ARMS  =  at-risk mental state; IQ  =  intelligence quotient; GAF  =  global assessment of functioning; PANSS  =  positive and negative syndrome scale; CAARMS  =  comprehensive assessment of at-risk mental states; HAM-A  =  Hamilton anxiety rating scale; HAM-D  =  Hamilton depression rating scale; BPRS  =  brief psychiatric rating scale.

a The CAARMS score is an overall score that was calculated by summing all subscales.

### Neuropsychological assessment

All participants were evaluated for response inhibition ability using the stop-signal task (SST), which was chosen from the Cambridge Computerized Neuropsychological Tests (CANTAB, CeNes Plc, Cambridge, UK). The SST is based on the “dual race model” [39] and gives a measure of an individual's ability to inhibit an ongoing motor response (stop-signal reaction time, SSRT). The test consists of two parts; in the first part, subjects are required to press a button on the left or right that is congruent with the direction of an arrow. In the second part, subjects perform the same task, but if they hear an auditory signal (a beep), they are instructed to withhold their response. Each subject completed five blocks of 64 trials each. Each block was divided into four sub-blocks of 16 trials for analysis. Every sub-block contained 12 “go” trials with no auditory stop signal and four “stop” trials that included an auditory signal presented following the stop-signal delay (SSD) period. The timing of the auditory stop signal changed throughout the test, depending on the subject's past performance, such that each subject was able to correctly withhold their response on approximately 50% of the trials. At the end of every assessed block, a feedback screen showed a graphical representation of the subject's performance; and the test administrator explained this representation to the subjects and also encouraged them to perform the tasks quickly. This test has five outcome measures: (a) the mean SSRT, which is the average time at which the subject was able to successfully inhibit the prepotent motor response; (b) the mean RT on go trials, which was the average time elapsed until the subject pressed the button on the press pad when there was no stop signal; (c) direction errors, which were instances of pressing the wrong button in both stop and go trials; (d) the proportion of successful stops, which refers to the number of times the subject successfully inhibited a response divided by the total number of stop signals; and (e) the SSD, which was the time at which subjects were able to correctly stop their response in 50% of trials.

### Intra-individual variability (IIV)

The SST consists of 20 sub-blocks composed of the same stimuli; thus, it is suitable for measuring IIV across sessions. IIV was evaluated by calculating the standard deviation of the twenty sub-blocks in the task across the three groups. The main variables of interest were the individual standard deviations for the SSRT and for the go-signal reaction time (go RT) and the mean SSRT, which represents mean performance in the stop process. The individual standard deviations reflect the dispersion of each sub-block in the stop and go processes for each individual subject. The equation applied to the computation of IIV is a straightforward standard deviation calculation, as follows:
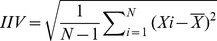
where 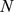 is the total number of blocks; 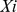 is the individual value of each block; and 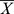 is the mean of all the blocks.

### Statistical analysis

One-way analysis of variance (ANOVA) was used to test for group differences in demographic characteristics (age, education, and IQ), intra-individual variability, SSRT, and other measures of task performance. The *chi*-squared test was used to test for between-group sex differences. Independent *t*-tests were used to measure differences between ARMS subjects and schizophrenia patients on clinical variables. Pearson's correlation coefficients were used to measure the associations of IIV and SSRT with psychotic symptoms in schizophrenia patients and ARMS subjects. The effect size was evaluated using the partial *eta* squared (*η* ^2^). To avoid the problem of multiple comparisons, Bonferroni corrections were applied. Significance levels were set at *p* values less than 0.05, divided by the number of comparisons (*p*<0.05: Bonferroni corrected *p* = 0.05/5 = 0.01).

## Results

### Demographic and clinical characteristics

The demographic and clinical characteristics of the three groups are shown in Table 1. No significant differences in sex, age, or educational level were found among the three groups. The IQ of the schizophrenia patients was significantly lower than that of the normal controls and ARMS subjects (*F*(2, 99)  = 8.90, *p*<0.001). The GAF scores of ARMS subjects and schizophrenia patients were significantly lower than those of normal controls (*F*(2, 99)  = 349.77, *p*<0.001). No statistically significant differences in psychotic symptoms were found between ARMS subjects and schizophrenia patients. However, HAM-D (*t* = −2.093, *p* = 0.041) and HAM-A scores (*t* = −2.094, *p* = 0.042) were higher in ARMS subjects than in schizophrenia patients.

### Intra-individual variability (IIV) and stop-signal reaction time (SSRT)

**Figure 1:**
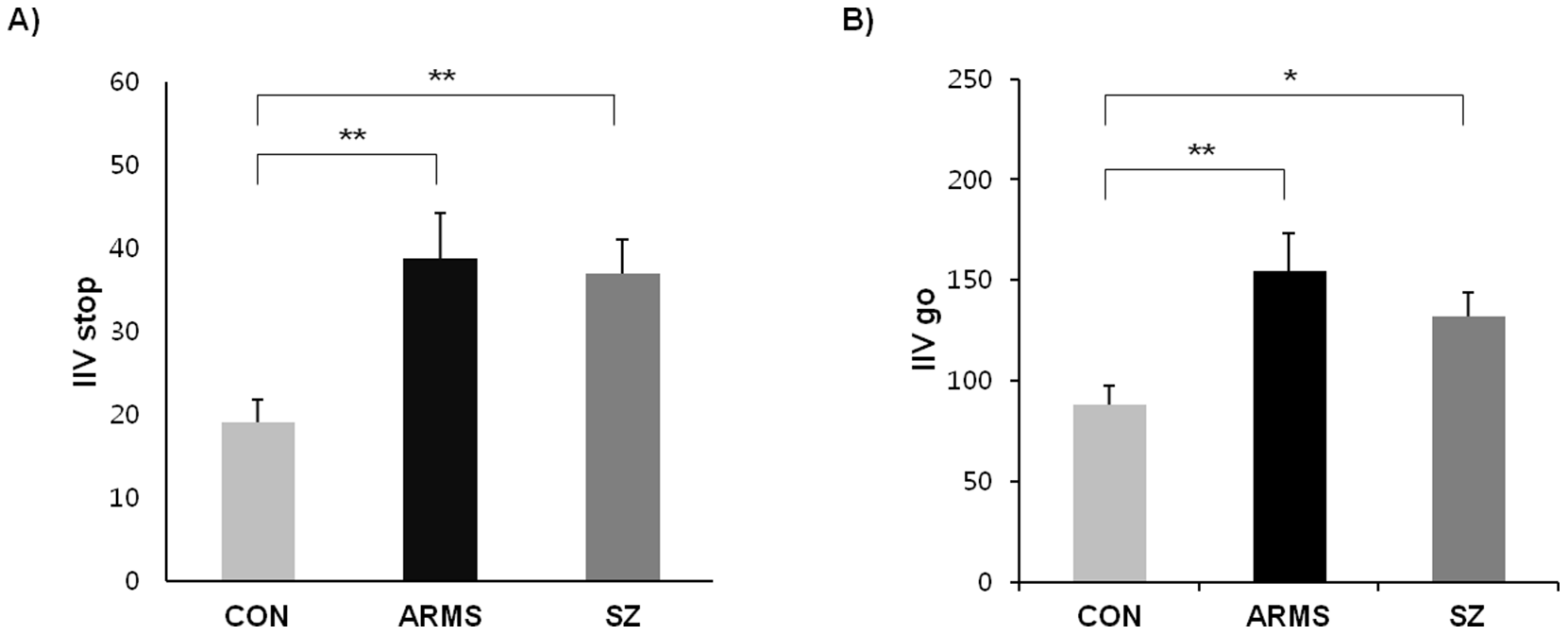
Mean performances of normal controls, ARMS subjects and schizophrenia patients on stop-signal task. A) Intra-individual variability (IIV) in stop process; B) Intra-individual variability (IIV) in go process. *Note.* CON  =  control; ARMS  =  at-risk mental state; SZ  =  schizophrenia. *p<.05. **p<.01.

**Figure 2:**
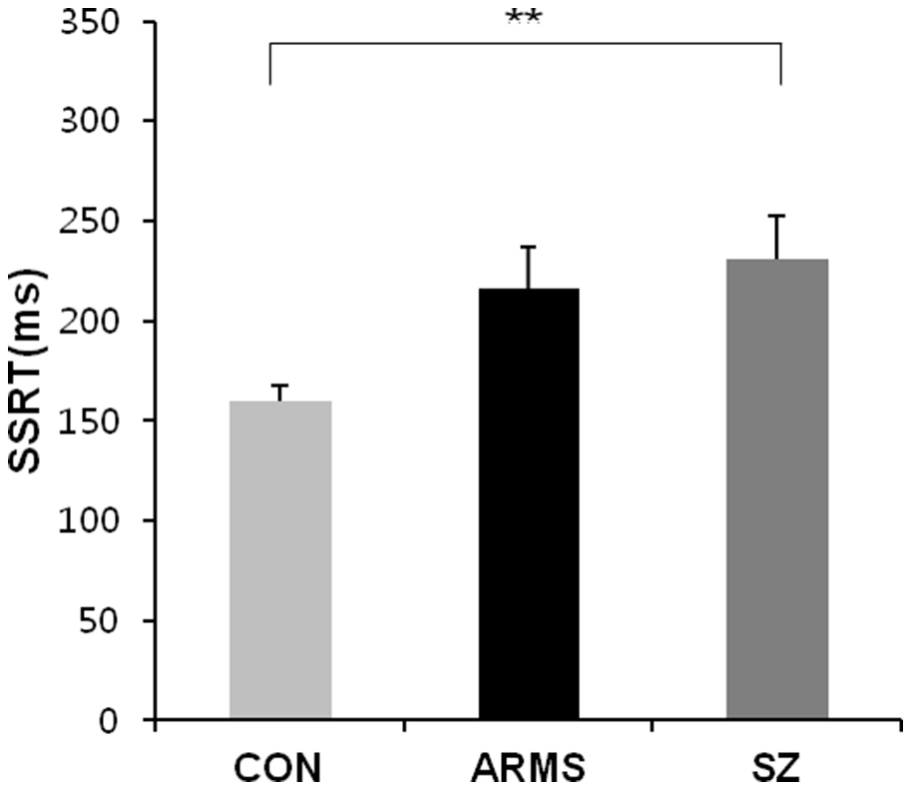
Mean performances in stop-signal reaction time (SSRT) of normal controls, ARMS subjects and schizophrenia patients on stop-signal task. *Note.* CON  =  control; ARMS  =  at-risk mental state; SZ  =  schizophrenia. **p<.01.

The means and standard deviations of the neuropsychological variables for ARMS subjects, schizophrenia patients, and normal controls are presented in Table 2. As shown in Figure 1, an ANOVA revealed a significant difference among the three groups in the IIV in the stop process (*F*(2, 99)  = 7.574, *p* = 0.001, *η* ^2^ = 0.13). *Post hoc* analysis revealed that the IIV in the stop process was increased in ARMS subjects (*p* = 0.004) and schizophrenia patients (*p* = 0.004) relative to normal controls. An ANOVA for IIV in the go process also revealed a significant effect of group (*F*(2, 99)  = 6.778, *p* = 0.002, *η* ^2^ = 0.12). *Post hoc* analysis indicated that the IIV in the go process was increased in ARMS subjects (*p* = 0.002) and schizophrenia patients (*p* = 0.038) relative to normal controls. Moreover, as shown in Figure 2, an ANOVA for SSRTs revealed a significant difference among the three groups (*F*(2, 99)  = 5.321, *p* = 0.006, *η* ^2^ = 0.1). *Post hoc* analysis indicated that SSRTs were slower in schizophrenia patients than in normal controls (*p* = 0.007). However, in the analysis of mean performance, the performance of ARMS subjects in the SSRT was not significantly different from that of normal controls. A main effect of group on go RT was observed at the trend level (*F*(2, 99)  = 4.415, *p* = 0.015, *η^2^* = 0.08). However, the ARMS subjects and schizophrenia patients did not differ with regard to the IIV in the stop and go processes, SSRTs and go RTs. No group differences were found in the proportion of successful stops (*F*(2, 99)  = 0.651, *p* = 0.524). The IQ score of the schizophrenia group was significantly lower than that of normal controls and ARMS subjects. However, the correlation analysis revealed no effects of IQ on any stop-signal task measures in the schizophrenia group.

**Table 2:** Mean performance on the stop-signal task in normal controls, ARMS subjects and schizophrenia patients; these means were examined using ANOVAs.

	Control	ARMS	Schizophrenia	Statistics	
	(n = 38)	(n = 27)	(n = 37)	*F*	*p-value*
IIV go	87.79 (59.28)	154.65(96.11)	132.06(72.99)	6.78	0.002 a,b
SSRT	159.46(48.17)	216.01(108.02)	231.11(127.90)	5.32	0.006 a,b
Go RT	459.65(143.20)	561.01(173.93)	547.82(152.18)	4.42	0.015 a
PSS	0.51(0.09)	0.55(0.16)	0.54(0.16)	0.65	0.524

*Note.* Data are presented as the means (SD). ARMS  =  at-risk mental state; IIV  =  intra-individual variability; SSRT  =  stop-signal reaction time; Go RT  =  reaction time on go trials; PSS  =  proportion of successful stops.

a *p*<0.05 for two-tailed tests.

b *p*<0.01 adjusted significance for two-tailed tests with application of Bonferroni correction for multiple comparisons.

### Associations of IIV and SSRT with psychotic symptoms

**Figure 3:**
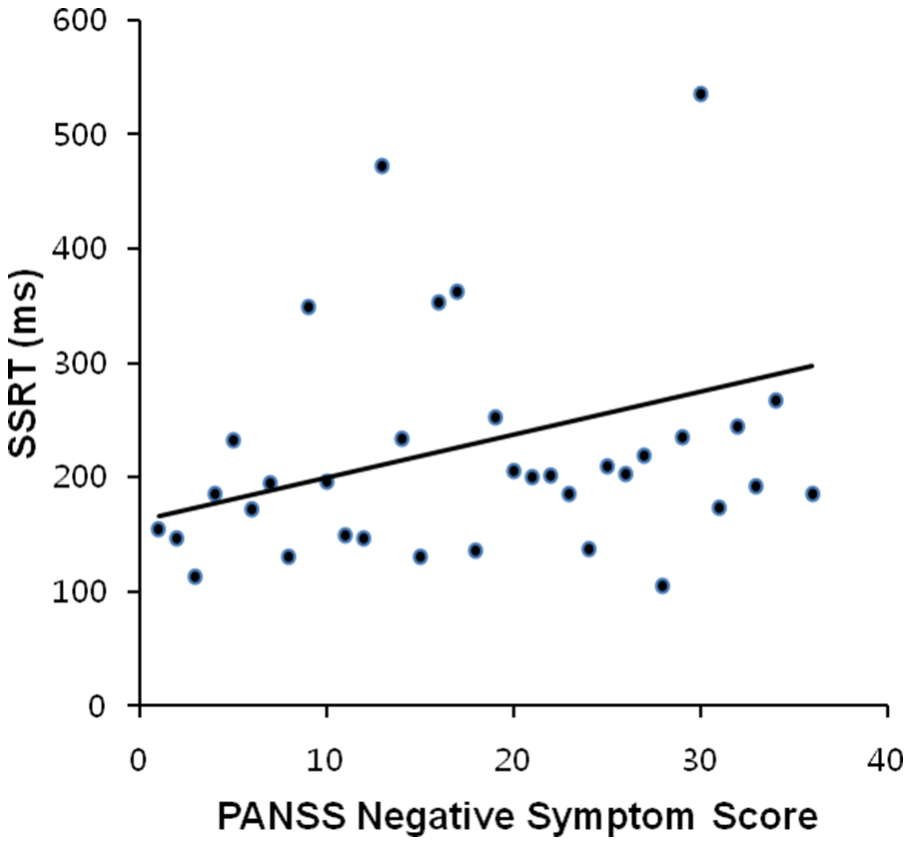
Relationship between stop-signal reaction time (SSRT) and severity of negative symptoms in schizophrenia patients. Pearson's correlation coefficient (*r*)  = 0.405, *p* = 0.014.

As shown in Figure 3, the association of IIV and SSRT with psychotic symptoms was assessed in schizophrenia patients and ARMS subjects. Impaired SSRT was related to the severity of negative symptoms in schizophrenia patients (*p* = 0.014); patients with increased negative symptoms required a greater amount of time to inhibit ongoing responses. Furthermore, the general psychopathology scores were positively correlated at the trend level with IIV in the stop process in schizophrenia patients (*p* = 0.063) (i.e., patients with increased general symptoms exhibited higher IIV). Positive symptom scores were not correlated with any other behavioral measure in the schizophrenia or ARMS groups. The positive and negative symptom subscales and the overall score on the CAARMS in ARMS subjects were not significantly correlated with IIV or SSRT.

## Discussion

To our knowledge, this study is the first to investigate the characteristics of intra-individual fluctuations in cognitive functioning in ARMS subjects. We observed increased IIV in ARMS subjects and schizophrenia patients relative to normal controls in both the stop and go processes. Interestingly, mean performance in the stop process did not differentiate the ARMS subjects from normal controls or schizophrenia patients. These findings indicate that both ARMS subjects and schizophrenia patients exhibit less stable cognitive processing than controls during response inhibition and response execution. Additionally, our findings suggest that IIV provides information regarding the cognitive and clinical features of ARMS subjects that is not detectable using conventional average performance measures.

Evidence from neurocognitive studies of ARMS has shed light on the developmental course of the illness. Two recent meta-analyses revealed small to moderate deficits across several cognitive domains in individuals at high risk for psychosis [5], [40], which indicates that the neuropsychological status of high-risk individuals was intermediate between that of normal controls and schizophrenia patients [3], [41]. In addition, Frommann et al. (2010) observed cognitive deficits in an early prodromal phase and found that further deterioration may follow in a later prodromal phase [42]. In particular, executive control dysfunction was found to emerge in the early prodromal state [42]. The present study shows that ARMS subjects exhibit increased IIV relative to normal controls even though their mean performance in the stop process did not differ significantly from that of healthy controls. These results suggest that increased IIV may present before deficits in mean performance. In addition, as IIV is thought to be associated with executive control processes that maintain the consistency of task performance, the increased IIV of ARMS subjects may manifest in the early prodromal state. Thus, IIV may be useful for predicting psychosis and developing early intervention strategies.

Recent investigations have delineated plausible neural underpinnings of behavioral variability. Increased variability is most strongly associated with frontal lobe regions because executive control processes are required to maintain task performance [15], [16], [17], [18]. Neuroimaging studies of ARMS subjects have identified abnormal frontal lobe processing during cognitive tasks [28] and structural abnormalities in frontal regions [43]. It has also been proposed that the inability to maintain consistency in a cognitive task may reflect endogenous brain structure and function, including individual differences in the integrity of functional brain networks [12] and the efficacy of neurotransmitter systems [44]. In particular, increased variability in schizophrenia may result from abnormal prefrontal activation and altered striatal DA function [25]. Recent studies have suggested that DA overactivity, which is associated with neurocognitive dysfunction [30], predates the onset of schizophrenia [29], [30]. Considering these findings, the unstable response patterns of ARMS subjects may originate from abnormal processing related to DA neuromodulation and to abnormalities in neural systems, such as those in the frontal lobe. Additional studies are needed to delineate the neural correlations underlying increased IIV in ARMS.

Executive dysfunction is one of the most common findings across studies of schizophrenia patients [45], [46] and ARMS subjects [2], [3], [5]. Response inhibition, one of the executive control processes, refers to the ability to suppress responses that are no longer required or are inappropriate in the context of ever-changing environments [47]. A key paradigm for the investigation of response inhibition is the stop-signal paradigm [48]. SSRT is the main dependent variable in stop-signal experiments and has proven to be an important measure of the cognitive control processes involved in stopping. Generally, SSRT has been found to be longer in patients with schizophrenia than in normal controls [49], [50], [51]. Our results are consistent with previous findings showing that SSRT is impaired in schizophrenia patients [49], [50], [51]; that is, schizophrenia patients need a greater amount of time to inhibit ongoing responses relative to normal controls. Longer SSRT was associated with increased severity of negative symptoms in schizophrenia patients, which indicates that patients with increased negative symptoms require more time to suppress responses relative to normal controls, which is consistent with the previous findings [51]. ARMS subjects exhibited SSRT values between those of controls and schizophrenia patients. These results indicate that although response inhibition in ARMS subjects is relatively preserved relative to schizophrenia patients, deficits in this domain may occur before the onset of psychosis and may become progressively worse over the course of the illness.

The limitations of the present study should be considered. First, all the schizophrenia patients except for 6 patients were receiving treatment with low dose of antipsychotics at the time of testing, and this might have affected the performance. Therefore, we could not exclude the possibility of medication effect on IIV and SSRT in schizophrenia patients. Second, although the current study suggests that the functional integrity of brain networks and dopamine-modulation processes are involved in the increased IIV in UHR individuals, we did not explore IIV on a neural level. Additional studies are needed to delineate the neural mechanisms associated with increased IIV in UHR individuals.

In summary, we conclude that increased IIV may present before cognitive deficits that are detectable by mean measures of performance in the prodromal phase of schizophrenia. Our findings highlight the importance of considering increased IIV as a behavioral manifestation of ARMS because the IIV of cognitive processing may serve as a more sensitive index for detecting intrinsic impairment than mean-level performance. Understanding the early behavioral signs of ARMS subjects who have not been explicitly identified may contribute to our knowledge of the underlying pathophysiological mechanisms of schizophrenia. Further studies of variability are needed to confirm and extend our findings in a variety of cognitive domains in ARMS subjects. Additionally, the sensitive changes in response inhibition IIV observed in ARMS subjects should aid the identification of behavioral markers that can be used for detecting earlier phases of the prodromal state.
