# Correction: Increased Intra-Individual Variability of Cognitive Processing in Subjects at Risk Mental State and Schizophrenia Patients

**DOI:** 10.1371/journal.pone.0155573

**Published:** 2016-05-10

**Authors:** Ye Seul Shin, Sung Nyun Kim, Na Young Shin, Wi Hoon Jung, Ji-Won Hur, Min Soo Byun, Joon Hwan Jang, Suk Kyoon An, Jun Soo Kwon

Table 2 appears incorrectly in the published article. Please see the correct Table 2 and its caption here.

**Table 2:** Mean performance on the stop-signal task in normal controls, ARMS subjects and schizophrenia patients; these means were examined using ANOVAs.

	Control(n = 38)	ARMS(n = 27)	Schizophrenia(n = 37)	Statistics	
				*F*	*P*
IIV stop	19.04(17.32)	38.70(28.54)	36.90(24.79)	7.57	0.001 ^a^^,^^b^
IIV go	87.79 (59.28)	154.65(96.11)	132.06(72.99)	6.78	0.002 ^a^^,^^b^
SSRT	159.46(48.17)	216.01(108.02)	231.11(127.90)	5.32	0.006 ^a^^,^^b^
Go RT	459.65(143.20)	561.01(173.93)	547.82(152.18)	4.42	0.015 ^a^
PSS	0.51(0.09)	0.55(0.16)	0.54(0.16)	0.65	0.524

*Note*. Data are presented as the means (SD). ARMS = at-risk mental state; IIV = intra-individual variability; SSRT = stop-signal reaction rime; Go RT = reaction time on go trials; PSS = proportion of successful stops.

^a^ p < 0.05 for two-tailed tests.

^b^ p < 0.01 adjusted significance for two-tailed tests with application of Bonferroni correction for multiple comparisons.
